# Features of Circulating Parainfluenza Virus Required for Growth in Human Airway

**DOI:** 10.1128/mBio.00235-16

**Published:** 2016-03-15

**Authors:** Laura M. Palermo, Manik Uppal, Lucy Skrabanek, Paul Zumbo, Soren Germer, Nora C. Toussaint, Bert K. Rima, Devra Huey, Stefan Niewiesk, Matteo Porotto, Anne Moscona

**Affiliations:** a Departments of Pediatrics, Microbiology and Immunology, and Physiology and Cellular Biophysics, Columbia University Medical Center, New York, New York, USA; b Applied Bioinformatics Core, Department of Physiology and Biophysics, Weill Medical College of Cornell University, New York, New York, USA; c New York Genome Center, New York, New York, USA; d Center for Infection and Immunity, Queens University, Belfast, Northern Ireland, UK; e Department of Veterinary Biosciences, College of Veterinary Medicine, Ohio State University, Ohio, USA

## Abstract

Respiratory paramyxoviruses, including the highly prevalent human parainfluenza viruses, cause the majority of childhood croup, bronchiolitis, and pneumonia, yet there are currently no vaccines or effective treatments. Paramyxovirus research has relied on the study of laboratory-adapted strains of virus in immortalized cultured cell lines. We show that findings made in such systems about the receptor interaction and viral fusion requirements for entry and fitness—mediated by the receptor binding protein and the fusion protein—can be drastically different from the requirements for infection *in vivo*. Here we carried out whole-genome sequencing and genomic analysis of circulating human parainfluenza virus field strains to define functional and structural properties of proteins of circulating strains and to identify the genetic basis for properties that confer fitness in the field. The analysis of clinical strains suggests that the receptor binding-fusion molecule pairs of circulating viruses maintain a balance of properties that result in an inverse correlation between fusion in cultured cells and growth *in vivo*. Future analysis of entry mechanisms and inhibitory strategies for paramyxoviruses will benefit from considering the properties of viruses that are fit to infect humans, since a focus on viruses that have adapted to laboratory work provides a distinctly different picture of the requirements for the entry step of infection.

## IMPORTANCE

Mechanistic information about viral infection—information that impacts antiviral and vaccine development—is generally derived from viral strains grown under laboratory conditions in immortalized cells. This study uses whole-genome sequencing of clinical strains of human parainfluenza virus 3—a globally important respiratory paramyxovirus—in cell systems that mimic the natural human host and in animal models. By examining the differences between clinical isolates and laboratory-adapted strains, the sequence differences are correlated to mechanistic differences in viral entry. For this ubiquitous and pathogenic respiratory virus to infect the human lung, modulation of the processes of receptor engagement and fusion activation occur in a manner quite different from that carried out by the entry glycoprotein-expressing pair of laboratory strains. These marked contrasts in the viral properties necessary for infection in cultured immortalized cells and in natural host tissues and animals will influence future basic and clinical studies.

## INTRODUCTION

Acute respiratory infection is the leading cause of mortality in young children under 5 years of age and accounts for nearly 20% of childhood deaths worldwide each year (1). Paramyxoviruses, particularly respiratory syncytial virus, human metapneumovirus, and the human parainfluenza viruses (HPIVs), cause the majority of childhood croup, bronchiolitis, and pneumonia (2). HPIV3 is widely prevalent in children: at least 60% of children have been infected with HPIV3 by 2 years of age, with 80% infected by 4 years of age, and in the United States, HPIV3 accounts for around 11% of pediatric respiratory hospitalizations. There are currently no vaccines or effective treatments for the HPIVs (2, 3).

HPIV3 possesses a single-stranded, nonsegmented, negative-sense RNA genome of approximately 15 kb in size. The virus infects its target cells by the coordinated action of the receptor binding protein hemagglutinin-neuraminidase (HN) and the fusion (F) glycoprotein, which together comprise the molecular fusion machinery. The first step of infection, entry of virus into the target cell, is initiated by attachment of HN to sialic acid-containing receptor molecules on the cell surface. Attachment starts with engagement of HN’s primary sialic acid binding site, which also possesses neuraminidase, or receptor cleaving, activity. Once the virus is receptor bound, HN activates the viral fusion protein (F) to a fusion-ready state, permitting its hydrophobic fusion peptide to insert into the target membrane. After F has inserted, it undergoes a regulated sequence of structural transitions leading to association between heptad repeats at the C terminus and N terminus of the molecule (HRC and HRN, respectively) (4) and culminating in merger of the viral and cellular membranes (5).

Circulating human HPIV3 viruses bear HN/F fusion pairs that are well suited to the natural host environment and that are different from those of the viruses that have until now served as models for fundamental research studies. We found that viruses bearing fusion (HN/F) machinery advantageous for growth *in vitro* feature different molecular determinants than those required *in vivo* (6). HPIV3 strains with a less active fusion machinery, or with briefer receptor engagement consequent to either lower receptor avidity or higher neuraminidase activity, are more successful in the natural host than viruses with more avid or longer receptor engagement (6). Our study of one clinically circulating HPIV3 isolate revealed an attachment/entry mechanism that is more stable and less readily activated for fusion than that of viruses adapted to growth in culture and also more sensitive to fusion-inhibitory molecules. The clinical strain also possessed greater receptor cleavage activity and lower receptor avidity—a balance between receptor binding and cleavage that favors short-term receptor engagement. Highly active fusion mediated by the viral glycoproteins seemed most likely to be not an advantage, but a detriment, in the natural host (7).

We propose that the HN/F fusion machine is tailored to the natural host. Circulating HPIV3 viruses bear HN/F fusion pairs that are well suited to the natural environment and reflect the physiologically relevant relationships between the properties of HN and F. Identification of the genetic basis for properties that confer fitness in the field will point to the critical viral structures essential for virus-host interplay during viral entry. Future development of entry inhibitors for respiratory viruses must also take into account the structural and functional features essential for fitness in humans.

Here we assessed the sequence diversity of HPIV3 clinical strains by determining the sequences of 8 human clinical strains. We characterized the molecular evolution of both the HN and F genes in an expanded panel of HPIV3 clinical isolates in a manner that preserves the integrity of the viral fusion machinery. To accomplish this, we passaged virus only in human airway epithelium (HAE), a tissue explant model that we have shown to faithfully represent the natural host target for HPIV (6–8). In addition we assessed the molecular evolution and genetic diversity of HPIV3 clinical isolates grown in HAE. We correlate the sequence data for the HN and F genes with quantitative measures of the HN/F functions of receptor avidity, neuraminidase, and fusion promotion. The results help specify the flexibility and constraints of the HPIV3 fusion machinery *in vivo* and define features important for infection in humans.

## RESULTS

### Stability of HPIV3 clinical strain sequences during passage on HAE.

The viruses that have been used for HPIV3 research in general are laboratory-adapted strains that efficiently fuse cultured monolayer cells. We found that these HPIV3 laboratory strains do not grow *in vivo* (8) and, conversely, that an HPIV3 clinical isolate (CI) that causes human disease has an HN/F pair poorly suited to fusion of immortalized cultured cells (6). In human airway epithelium (HAE)—which provides a good working model for the natural host target for HPIV3 (8, 9)—we found that the clinical isolates grow at high titer (6) (see Fig. 6). To characterize the molecular evolution of HN and F genes in an expanded panel of HPIV3 clinical isolates, it was essential to propagate them without adapting them to laboratory conditions in immortalized cells—in order to preserve the integrity of the viral fusion machinery. We therefore assessed whether propagation of CIs in HAE preserves the features of the isolates or also results in adaptation, as evidenced by alterations in the genome. The isolates of HPIV3 assessed here were isolated from patients and delivered from the hospital clinical microbiology laboratory as direct patient specimens with no prior manipulation.

**FIG 1:**
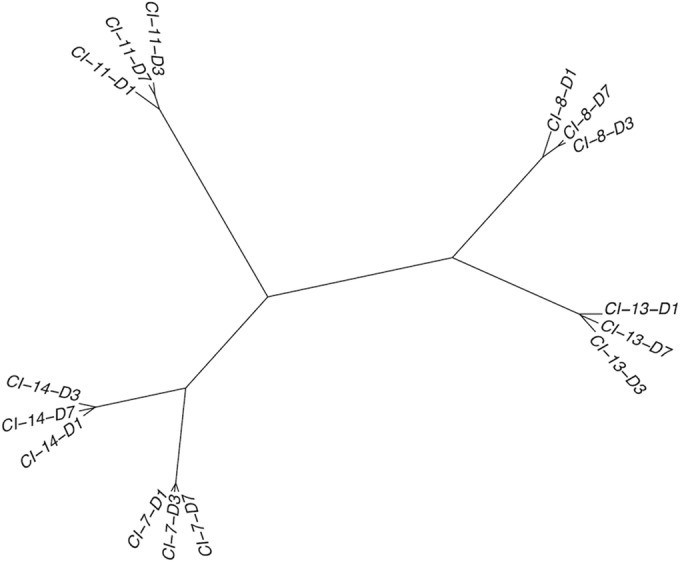
Neighbor-joining tree of Euclidean distances between clinical isolates (CIs) sequenced directly from HAE supernatant fluids. In the nomenclature format shown, “CI-11-D3,” for example, represents CI-11 at day 3 of collection. Distances between genomes were calculated based on the relative frequencies of alleles at each position that was covered by at least 10 reads

HAE were infected with CI-7 through -14, virus samples were harvested as described in Materials and Methods on days 1, 3, and 7, and the genome of each harvested sample was sequenced. We were unable to construct day 1 consensus genomes for CI-9, -10, and -12 due to poor sequencing depth, and therefore, these isolates were excluded from this portion of the analysis. The change in sequence identity across CI-7, -8, -11, -13, and -14 on each day was quantified by computing the Euclidean distances between each genome. A neighbor-joining tree of these distances shows that each isolate maintains a high degree of nucleotide identity during passage in HAE (Fig. 1).

The proportions of individual single-nucleotide polymorphisms (SNPs) in each open reading frame (ORF) of CI-13 are shown in Table 1. CI-13 was used for the SNP analysis to analyze the stability during passage in HAE because it represents a CI with highly reliable sequence coverage and is an isolate with high variability over the days of growth relative to this group of viruses. From day 1 to day 7, the minor variants generally decrease in proportion, and the viruses appear to converge to more uniform sequences during passage in HAE. This convergence is also seen in CI-7, -8, -11, and -14. Inspection of individual ORFs reveals that most SNPs are found in the NP and L genes. This pattern is also exhibited by CI-7, -8, -11, and -14. Each contains a few SNPs in the NP gene, clustered between positions 380 and 450. In addition, these isolates feature on average 23 SNPs across the L gene. These SNPs are more uniformly distributed, with two distinct ranges featuring most changes. These are the regions spanning base pairs 9000 to 10600 and base pairs 13000 to 15000. The isolates also tend to feature a cluster of SNPs in the terminal region of the gene from base pairs 15200 to 15400, but it should be noted that sequencing depth is relatively poor in this region. The HN and F ORFs feature few, if any, minor variants and only in small proportions. The absence of minor variants in HN and F across the 7 days indicates that there is little, if any, alteration to the fusion machinery of HPIV3 when these viruses are grown in HAE. Figure 1 indicates that the passage of the clinical strains on HAE does not alter the viruses in any significant way. After growth for 7 days on airway epithelium, the identity of each strain is largely preserved. Isolates from the same patient cluster closely together, regardless of the day of collection.

**TABLE 1:** SNP frequencies during passage of HPIV3 strain CI-13

Position (nt)	Major variant	Minor variant	ORF	Coding difference	Kinetics	SNP frequency (%) on:		
						Day 1	Day 3	Day 7
396	G	T	NP	V96F	D	18	5	2
398	C	T	NP	V96F	D	18	0	1
400	A	G	NP	K97R	D	8	0	0
403	A	T	NP	Y98F	D	7	0	0
407	C	T	NP	None	D	7	0	0
409	T	A	NP	I100K	D	7	0	0
414	A	T	NP	M102L	D	7	5	0
430	T	A	NP	L107Q	I	0	4	0
438	C	T	NP	Q110*	D	13	4	0
439	A	G	NP	Q110*	D	13	0	0
459	G	T	NP	V117F	D	29	27	9
462	A	G	NP	K118E	D	28	26	8
1967	T	C	P	C62R	I	0	7	0
1967	T	C	C	None	I	0	7	0
5404	T	A	F	F111L	I	1	4	0
7620	C	G	HN	P272R	D	3	0	0
10585	A	T	L	D647V	D	10	0	0
13811	G	A	L	None	D	8	0	1
13934	T	G	L	None	D	7	5	1
13999	T	G	L	L1785R	D	7	2	0
14247	G	T	L	V1868F	D	8	0	0
14912	C	T	L	None	D	10	0	2
15042	T	A	L	S2133T	D	9	0	1
15227	C	A	L	None	I	0	7	1
15273	C	A	L	P2210T	D	7	0	0
15277	G	A	L	R2211K	I	0	11	0
15288	G	A	L	E2215K	I	0	4	0
15292	C	A	L	P2216H	I	0	9	0
15294	G	A	L	E2217K	I	0	4	2
15314	C	A	L	N2223K	I	0	11	2
15317	C	A	L	Y2224*	I	0	12	0
15324	C	A	L	H2227N	D	10	0	2
15338	T	A	L	D2231E	D	9	0	0
15342	G	A	L	D2233K	D	10	0	0
15344	T	A	L	D2233K	D	10	0	0

### Sequence relationships among HPIV3 clinical strains and lab-adapted strains.

**FIG 2:**
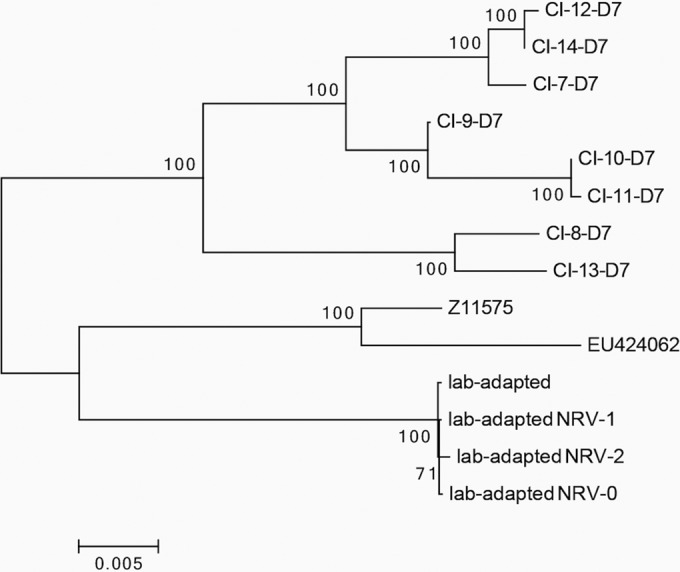
Maximum likelihood phylogenetic tree derived from an alignment of 14 complete HPIV3 genome sequences. Bootstrap confidence values of >70% (from 500 tests) are indicated. The lab-adapted strains used here are the original laboratory strain (34) and three neuraminidase-resistant variants (NRVs) selected for resistance to exogenous neuraminidase: NRV-0 (12), NRV-1 (8, 10), and NRV-2 (11).

In order to determine how similar clinically circulating strains of HPIV3 are to well-characterized lab-adapted strains, we generated a phylogenetic tree based on an alignment of 14 genome sequences (Fig. 2). The tree includes the day 7 consensus sequences of all eight clinical isolates, as well as our previously characterized lab-adapted strains (10–12). Also included in the alignment is the JS strain (Z11575), a clinical isolate sequenced in 1991 and grown on immortalized cell lines (13), and the EU424062 strain, a JS mutant used in the alignment and in the construction of the consensus sequences of our clinical isolate genomes (14). The tree features two main clusters consisting of the clinical isolates and the lab-adapted strains, including the JS strain and its variant. These two clades diverge at the topmost node in the tree, indicating a clear divergence between the clinical isolates found *in vivo* and the various lab-adapted strains: our original laboratory strains and the three variants selected for resistance to exogenous neuraminidase (neuraminidase-resistant variants [NRV]), as well as Z11575 and EU424062. These data suggest, in agreement with what we have noted in the past, that the lab-adapted strains are not representative of HPIV3 clinically circulating strains.

### Sequence diversity and divergence among HPIV3 clinical strains.

**FIG 3:**
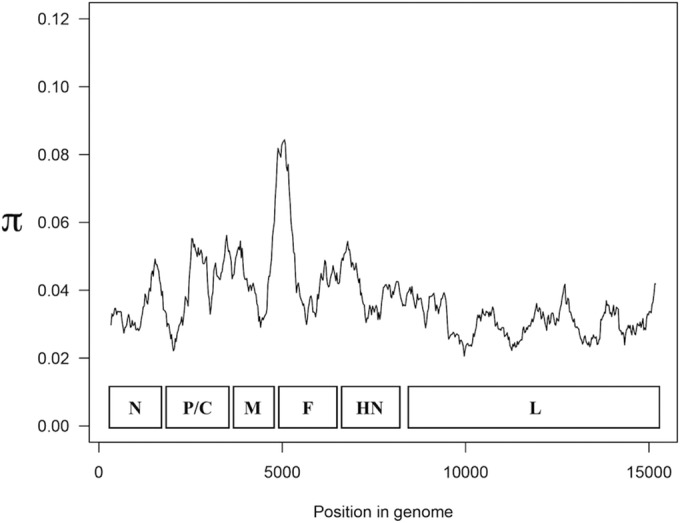
Nucleotide substitutions per site (π) measured in a 500-nt sliding window along an alignment of 14 complete HPIV3 genome sequences. Values are plotted to correspond to the center of the window. The arrangement of ORFs is indicated.

To identify sites within each gene that may relate to functional differences, we analyzed the extent of variation along the HPIV3 genome. The data presented in Fig. 2 were used to identify regions within each gene that may lack constraint. We computed the number of nucleotide substitutions per site, expressed as π (Fig. 3) (15). Peak nucleotide diversity occurs at nucleotide (nt) 5061, which is the midpoint of the window spanning from nt 4811 to nt 5311. This window consists of the last 20 nt of the M protein, the M-F intergenic region, and the first 221 nt of the F protein, which correspond to the first 73 amino acids (aa) of the F protein. Nucleotide diversity decreases further downstream, but π remains greater than 0.06 until nt 5236, which is inside the window from nt 4986 to 5486, with the latter corresponding to aa 132 of F. Thus, peak diversity in the HPIV3 genome occurs in the first portion of the F protein. Nucleotide diversity reaches its global minimum along the genome in the window from nt 9711 to 10211 in the L protein. The midpoint of this window corresponds to aa 432 of the L protein, suggesting that this region may be highly conserved, and this region indeed contains the INGYRxxHGGxWPP motif present in all morbilliviruses and respiroviruses (16).

**FIG 4:**
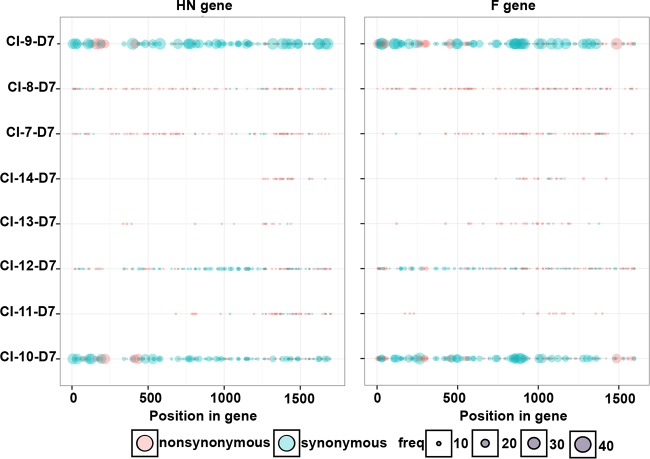
Locations of synonymous and nonsynonymous minor variants in the HN and F ORFs. Minor variants are depicted by their frequency and whether the nucleotide substitution resulted in a corresponding change in amino acid sequence. Each dot represents a single variant; however, some positions exhibited multiple minor variants, with each major allele-minor allele comparison resulting in a separate overlapping dot.

We also assessed diversity within the clinical isolates by quantifying the synonymous and nonsynonymous minor variants in each ORF of the day 7 consensus sequences (Fig. 4). To do so, we selected SNPs with at least 8 reads and at a frequency of at least 2% of total coverage. The clinical isolates feature little nucleotide diversity in the HN (Fig. 4, left panel) and F (Fig. 4, right panel) ORFs, with the exception of CI-9 and -10. Even in CI-9 and -10, the bulk of diversity consists of synonymous mutations, and thus there is a high degree of homogeneity at the amino acid level for each of these clinical isolates. The relatively large amount of nucleotide diversity in CI-9 and -10 may suggest a distinct subpopulation of HPIV3. The observations for HN and F are echoed in each of the other ORFs as well (data not shown). Of note, two residues of significant interest to us in functional experiments are highly conserved in all clinical isolates we studied and different from the laboratory strains. All CI coding sequences for HN predict an asparagine at position 556, where the laboratory-adapted strains have aspartic acid. All CI coding sequences for F predict a glutamic acid at residue 108—the cleavage activation site of F—where the laboratory strains have lysine. The potential significance of each of these residues is discussed below.

### Mode of HPIV3 evolution.

The analyses shown in Fig. 3 and 4 aimed to identify diversity at the gene level in an unbiased fashion; now we searched for specific sites where selective pressure may have operated. To assess the mode of HPIV3 evolution, we used the ratio of nonsynonymous to synonymous nucleotide substitutions (*dN*/*dS*) as an indicator of positive selection. Pairwise comparisons of the *dN*/*dS* ratio were made (Table 2). A *dN*/*dS* value of 0 indicates that nonsynonymous substitutions have been completely suppressed, a *dN*/*dS* value of 1 indicates that neutral accumulation of substitutions has occurred, and a *dN*/*dS* value of >1 indicates that positive selection may have taken place. The *dN*/*dS* values in each ORF are all below 1 (averaging 0.09), implying that purifying selection has taken place and that each gene has been subject to strong selective constraint. Table 2 also shows that the κ values, which denote the ratio of transitions over transversion mutations, are very high in this HPIV3 data set. The average κ value is 16.7, which is notably higher than those observed for other paramyxoviruses, such as 8.17 for PIV5 and 5.1 for measles virus (17). The significance of this preponderance of transitions is not clear but may reflect an intrinsic property of the HPIV3 polymerase.

**TABLE 2:** Values of *dN*/*dS* and κ in 9 HPIV3 genomes for individual ORFs

ORF	Size (nt)	*dN*/*dS*	κ
N	1,548	0.04211	12.31544
P	1,812	0.24920	14.25077
C	600	0.16507	12.28103
M	1,062	0.03169	29.71235
F	1,620	0.07461	13.99032
HN	1,719	0.06250	22.27403
L	6,702	0.02790	13.18412

We also tested for positive selection at individual amino acid residues in each ORF and checked for consistency across 3 different methods (Bayes empirical Bayes [BEB] in PAML, SLAC, and FEL). With the exception of residue 279 in the P protein, there is no consistent evidence of positive selection at individual residues at significance levels of *P* < 0.05 in PAML and *P* < 0.1 in SLAC and FEL (Table 3).

**TABLE 3:** Positively selected sites in each ORF as determined by the Bayes empirical Bayes, SLAC, and FEL methods

ORF	Positively selected site(s) by:		
	PAML (*P* < 0.05)^a^	SLAC (*P* < 0.1)	FEL (*P* < 0.1)
N	None	None	None
P	178, 258, 279, 328	None	279, 584
C	173	None	None
M	None	None	None
F	17	None	None
HN	138	None	None
L	None	None	2223

a Bayes empirical Bayes method implemented in PAML.

While the F, HN, C, and ORFs appear to have a few residues that have undergone selection according to PAML, these results are not consistent across methods. Therefore, these sites can likely be interpreted as false positives. However, position 279 in the P ORF may be a true instance of positive selection, as it was detected as a statistically significant residue by both the BEB and FEL methods.

### Functional analysis of CI HN and F genes.

In order to understand the selection forces that affect HPIV3 evolution in the natural host, we next analyzed the biological properties of the circulating HPIV3 strains requisite for viral entry and spread. Our previous work indicated that lung-adapted viruses carried less active HN/F fusion pairs. Here we characterized the function of HN and F derived from CIs. HNs derived from several different CIs were identical, so the eight viruses yielded five different HN sequences. The sequences of CI-7 (representing CI-7, CI-12, and CI-14), CI-8, CI-9, CI-10 (representing CI-10 and -11), and CI-13 HN were studied.

### Receptor avidity and receptor-cleaving activities differ in clinical isolate strains compared to laboratory-adapted strains.

HPIV3 enters cells by fusing directly their viral membrane with the cellular membrane. The HN and F proteins orchestrate this process upon receptor engagement. The HN protein performs four critical functions that are important for viral entry and spread: it stabilizes F before receptor engagement, mediates receptor binding, activates F, and cleaves the receptor (2, 10, 18, 19). Upon receptor engagement, HN activates the F protein to undergo a series of structural conformations that facilitate the direct fusion of both membranes. At later stages of the viral life cycle, the HN’s neuraminidase activity is required to mediate release of budding viruses from the infected cell.

The HNs derived from CIs were compared for their ability to bind and release sialic acid receptors. For measurement of HN receptor binding avidity, cells transiently expressing each of the HN variants were pretreated with neuraminidase to partially deplete receptors on the expressing cells’ surfaces. Receptor-bearing cells, in this case erythrocytes (RBCs), with different degrees of receptor depletion were added and used to quantify binding to the HNs (10, 20). HN molecules with higher avidity bind RBCs that have lower receptor density, so the level of RBC receptor depletion that still permits binding provides a measure for avidity. The HNs derived from CIs all showed similar avidity for sialic-acid containing receptors, with 50% of binding to RBCs treated with ~10 mU of neuraminidase, and the avidity of the CI HNs is lower than that of the lab-adapted strain (reference strain) HN, which showed 50% of binding to RBCs treated with 27.5 mU (Table 4). The receptor avidity of all of the CI strains is similar to that of the first CI we studied (6), suggesting that this is a general property of these strains.

**TABLE 4:** Characteristic of HPIV3 CI HN proteins

HN protein type	NA level (% of HN lab-adapted level)	Receptor avidity (mU for 50% RBC binding)
Lab adapted	100	27.5
CI-1	1,100	15.0
CI-3	618	10.0
CI-8	1,083	10.0
CI-10	1,819	10.0
CI-13	323	10.0

To compare levels of receptor cleavage, we analyzed the neuraminidase activity of the HN derived from these CIs compared with HN from the lab-adapted strain (Table 4). The HNs derived from CIs showed from 4- to 10-fold-higher neuraminidase activity than HNs derived from the lab-adapted strains. Overall, HNs derived from lung-adapted viruses have a ratio of neuraminidase to avidity of 0.4 to 1, indicating that these HNs are less likely to be engaged to sialic acid-containing molecules than lab-adapted derived HNs (ratio of ~0.04). We examined the aligned amino acid sequence of HN for commonality between CIs in the ectodomain. All CI HN amino acid sequences have an asparagine at position 556, where the lab-adapted strain has aspartic acid. We assessed the effect of this D556N alteration in HN on the neuraminidase activity of expressed laboratory strain HN and found that HNs bearing this residue have approximately 5-fold-greater neuraminidase activity than the lab-adapted strain HN (data not shown). These data suggest that the propensity for receptor cleavage, shared by all of the CIs we have studied, may be at least in part attributed to residue 556.

### Properties of the HN/F fusion machinery of HPIV3 CIs.

**FIG 5:**
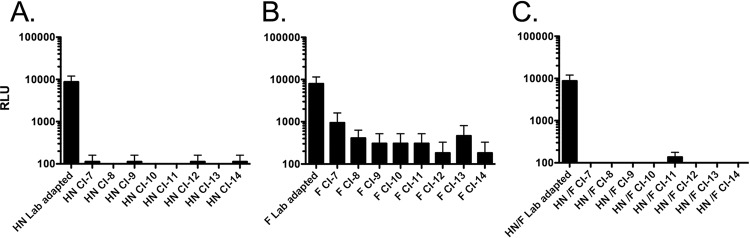
The CI-derived HN and Fs are less efficient at promoting cell-cell fusion than the proteins derived from HPIV3 virus adapted to growth in immortalized cells. (A) 293T cells coexpressing the indicated HN with the F derived from a laboratory reference strain, (B) 293T cells coexpressing the indicated F with the HN derived from a laboratory reference strain, or (C) 293T cells coexpressing the indicated HN/F pair were allowed to fuse with receptor-bearing cells at from 37°C on. Cell fusion was measured as relative light units (RLU [*y* axis, log scale]) in the presence of each HN/F pair (as indicated on the *x* axis). The bars represent means ± standard errors of the meant (SEM) from triplicate samples from a representative experiment.

We previously identified a single HPIV3 clinical isolate that carried a less efficient HN/F fusion pair. We showed that both the HN and the F of this isolate (CI-1) contributed to the reduced fusion feature (6). To determine the functional properties of the HN/F fusion pairs of the CIs studied here, HN and F genes derived from these CIs were cloned into expression vectors, and their fusion properties were analyzed using a quantitative β-galactosidase complementation fusion assay. The HN derived from each CI was analyzed in combination with F derived from a lab-adapted HPIV3 virus (Fig. 5A). Fusion promoted by each CI HN is significantly reduced compared to that promoted by the HNs from lab-adapted viruses (by more than a 2-fold log change). The F proteins derived from CIs also have reduced fusion competency compared to the fusion protein derived from the lab-adapted virus (Fig. 5B). Note that in order to assess fusion in immortalized cells mediated by the F proteins derived from CIs, the clones were mutagenized to introduce K at residue 108 in order to permit cleavage activation of F in immortalized cells. The expressed CI F proteins bearing K108 are efficiently cleaved in immortalized cell culture, as demonstrated on gel electrophoresis with expressed E108 CI F protein and expressed G108 laboratory strain F protein as controls (data not shown). Fusion is minimal when the CI-derived HNs and Fs are tested in pairs (Fig. 5C). These results are similar to our results for a single clinical isolate, indicating that the contribution of both HN and F to the decreased fusion mediated by lung-adapted HPIV3 strains is a general principle of circulating strains. The variant F protein carrying a mutation at position 108 (K108G) was used as a negative control in these experiments, since this variant F is not proteolytically cleaved in 293T cells and thus is not fusion competent.

### CIs are fit for growth *in vivo.*

**FIG 6:**
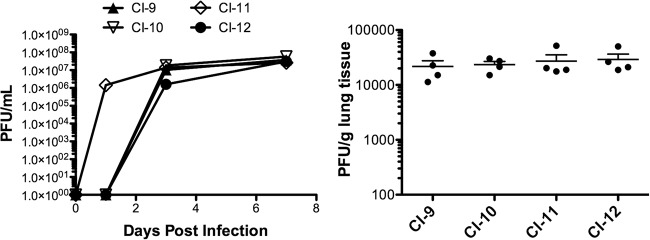
CI growth in HAE and cotton rats. (Left panel) HAE were apically infected with 4,000 PFU of the indicated HPIV3 CI. The virus released from the apical surface was collected at several time points. Viral titer (PFU per milliliter) was determined by plaque assay. Data points are means ± standard deviations (SD) from triplicate measurements and are representative of 3 experiments. (Right panel) Groups of 4 cotton rats were infected with the indicated HPIV3 CIs and sacrificed 3 days postinfection. Viral titer (PFU per gram of lung tissue) was determined by plaque assay.

To assess the growth of HPIV3 CIs in settings that resemble the natural host tissue, HAE cultures were infected apically, and viral production was measured on the indicated days after infection. Individual CIs showed slightly different kinetics in their growth curves at early time points. However, all of the CIs tested here reached similar maximal titers in HAE at day 3 postinfection, as we previously described for laboratory-adapted strains and a field isolate (6, 8), suggesting similar fitness for growth in HAE (Fig. 6, left panel).

HPIV3 CIs grow efficiently in cotton rats, a model that has been well validated for viral respiratory disease (21). Cotton rats (4 per group) were infected with CI-9, -10, -11, and -12. The animals were sacrificed 3 days postinfection, and the viral titers were measured by plaque assay. Day 3 was chosen because we have shown that HPIV3 titers peak in cotton rats at this time point, declining over the following days (21). CI viruses attained titers of 5 × 10^4^ PFU/g of lung tissue at day 3 postinfection, similar to those we had described for one field isolate (Fig. 6, right panel) (6) and approximately 1 log higher than the titers we have previously shown for laboratory-adapted strains in cotton rat lungs (8, 22, 23). These results indicate that regardless of the sample’s origin, CIs grown in HAE maintain their genomic sequences and retain their ability to efficiently infect cotton rats, making this model well suited to studying pathogenesis of field isolates and assessing new antiviral drugs.

## DISCUSSION

To understand and interfere with natural viral infection, it is critical to study the mechanisms that affect virus-cell interplay using field strains in the context of the specific host. Paramyxovirus research has, for convenience and technical reasons, relied on the study of laboratory-adapted strains of virus in immortalized cultured cell lines. We have shown, for parainfluenza viruses, that conclusions drawn from this experimental combination can be misleading with regard to the receptor interaction and viral fusion properties that govern entry and fitness *in vivo* (6). For another paramyxovirus, measles virus, laboratory-adapted strains and field strains use different cell surface receptors, highlighting the importance of this distinction: laboratory strains use CD46 (membrane cofactor protein) as an entry protein, but clinically relevant viruses use CD150/signaling lymphocyte-activation molecule (SLAM) or nectin-4 as the entry receptor (24). Here we interrogated the HN/F fusion machinery of HPIV3 in two settings that we propose represent “real life” for this virus: human airway epithelium (HAE) and cotton rats. Analysis of clinical strains suggests that the HN/F fusion pairs of circulating HPIV3 viruses maintain a balance of properties that result in an inverse correlation between fusion in cultured cells and growth *in vivo*. These results suggest that for HPIV to infect its host, fusion above a necessary threshold level is a detriment, not an advantage. The fusion pairs operate under constraints that govern viability in tissues, in an animal model, and in human beings.

For HPIV3 laboratory variants, we previously showed that increased fusion in cultured immortalized cells correlates with reduced viral titers *in vivo*, and growth of the HN variants *in vivo* ranks in the opposite order from their fusion of a monolayer of cultured cells (7, 8). The severity of lung pathology in cotton rats correlates with the growth of each variant *in vivo* (8, 22). We posit that the HN/F fusion pairs of field strains of HPIV3 (in contrast to laboratory-adapted strains) exhibit a relationship between binding, fusion promotion, and receptor cleavage that is a disadvantage for growth in immortalized cells—but confers fitness for growth *in vivo*.

Here we used whole-genome sequencing of field strains of HPIV3 isolated from humans to analyze the diversity and the commonalities of circulating strains of HPIV3 at the genomic level. The sequences were used to define functional and structural properties of proteins of circulating strains in order to identify the genetic basis for properties that confer fitness in the field. Functional experiments show that while laboratory-adapted and CI strains share the basic steps and properties of HN/F interaction, the balance between these properties at each step (e.g., F triggering versus receptor avidity) is different between CI and laboratory strains and is shared across all CIs studied. The CI fusion pairs behave similarly to each other, with lower net fusogenicity and decreased F activation compared to those of laboratory strains. Viral growth correlates inversely with fusogenicity of the viruses’ HN/F pairs and directly with the tendency of the pair to maintain F in its prefusion state.

On initiating these studies, a concern was that clinical samples could contain a heterogenous population of viruses that could be lost in HAE cells. To assess the homogeneity, we sequenced the genomes of CI HPIV3 at multiple time points as they were passaged in HAE cultures and analyzed them for changes over time. The HPIV3 CI maintained a high degree of nucleotide identity during passage in HAE. Several of the CIs showed convergence to a more uniform sequence during growth in HAE, supporting the contrast to laboratory strains and suggesting that—while this system is a closer mimic of the natural host than immortalized monolayer cell culture—growth in HAE may result in some selection compared to virus in a human host toward a common “lung-adapted/clinical” consensus. We propose that this approach be used to propagate and study authentic circulating strains, to avoid the emergence of mutations under the selective pressure of growth in cultured immortalized cells. As one example, in a recent study by Mizuta et al. (25), when clinical isolates of HPIV were passaged in immortalized cells in the course of amplifying them for sequencing studies, the passaged viruses contained the very mutations that we have shown to confer growth in immortalized cells (H552Q and N551D). These two mutations—in what we have shown to be a second sialic acid binding/fusion promotion site at the dimer interface of HPIV3 HN—confer increased fusogenicity on viruses bearing this HN and emerge in the selective pressure of growth in monolayer culture. We suggest that even relatively brief passage in the selective pressure of cultured cells alters HPIV3 properties.

One impact of this discovery may be on the approach to vaccination against HPIVs (and possibly other paramyxoviruses), where the generation of recombinant viral vaccines with engineered attenuation is showing promise (17). Recombinant vaccine preparation relies on immortalized cell lines for production of vaccine virus. Our results indicate that even brief growth in such cells selects for properties that may be disadvantageous *in vivo*, and this possibility should be considered early on in the process of assessing candidate vaccine viruses and designing the process for growth of the vaccine strains.

For antiviral development for potential clinical efficacy, it will be critical to evaluate candidates using clinical isolates in natural host tissues, rather than laboratory strains of virus in immortalized cells, as we have recently shown for lipid-conjugated antiviral peptides derived from the HRC region of HPIV3 F (6, 26). Of note, in the present study the HRC and HRN regions of F were found to be conserved between laboratory and clinical strains and do not seem to contribute to the observed phenotype. This suggests that peptide antivirals derived from these regions may be effective for a range of circulating HPIV strains.

The sequencing strategy we used in order to be able to sequence clinical strains directly without passage in immortalized cells does not rely on designing sequence-specific primers that may amplify only a subset of samples (in case of mutations). We obtained full genome sequence coverage from as few as an estimated 20,000 starting copies (determined as described in reference 27) and found that relatively uniform whole viral genome coverage can be achieved with 100,000 paired end reads, suggesting that samples could be sequenced in a single MiSeq run. Whole-genome viral sequencing from clinical samples should be both technically and economically feasible, permitting investigations to avoid the use of laboratory-adapted strains of virus in immortalized cultured cell lines.

The sequencing results we obtained, along with the comparison with functional data, help define the flexibility and constraints of the fusion pair and reveal specific requirements. All of the CIs are different from lab-adapted strains and segregate together at the genomic level (Fig. 2). Significant variation seems to be permissible in the first few residues of F, a region that is cleaved from the protein; variation at this region was also noted in the original HPIV3 sequencing studies of HPIV3 genes by Coelingh and Winter in 1990 (28). A region of high variation in the sequence of isolates of PIV5 encompassing the 3′ untranscribed region (UTR) of the M gene, the intergenic sequence, and the 5′ UTR of the F gene was identified by Rima et al. (29), implying a lack of constraint in this region for PIV5 as well.

In the course of assessing for evidence of positive selection across the entire genome of the CI, position 279 in the P ORF emerged as the only consistent positively selected residue during growth in HAE. It is also possible that the selection affects the alternate polypeptides that are encoded within the P gene, thereby affecting replication (30), and this is a subject of future investigation. The clinical isolates we assessed were all obtained from the same geographical location within 3 years of each other, and this may contribute to the close relationship between them: in the future, if clinical strains in other geographical locations are collected properly for study without passage in conventional culture, it will be possible to address difference in circulating HPIV3 globally and over time.

In this analysis, we made the striking finding that the F proteins of the CI are all different from the laboratory strains at position 108, the critical cleavage activation site residue of F. The F protein is synthesized as a single polypeptide chain that forms a trimer before being cleaved by host cell proteases to yield a membrane-distal and membrane-anchored subunit. The new N-terminal region of the membrane-anchored subunit contains the hydrophobic fusion peptide that inserts into target membranes during fusion (reviewed in reference 31). The specific host cell proteases required for cleavage activation of the F protein *in vivo* have not been identified. (This work is in progress.) Cleavage of F in immortalized monolayer cells is carried out by furin proteases (32), and it has been assumed that these also mediate cleavage *in vivo*. However, while the laboratory strains all bear an F with a K at position 108 that is cleaved during growth in immortalized cells, the CIs all bear an F with E at position 108 that is not cleaved in immortalized cells, and representative CI F proteins are predicted by cleavage site analysis to be resistant to furin cleavage (data not shown). Substitution of K at residue 108 in CI F proteins restored cleavage in immortalized cell monolayers (data not shown). However, CI HPIV3 released during growth in HAE bears a cleaved F (data not shown), confirming that the CI F is cleaved by proteases that are present in HAE and in vivo. Of note, other examples exist in the literature of E at the residue corresponding to 108 (28, 33); Coelingh and Winter (28) identified HPIV3 isolates with E at residue 108 among strains isolated from children; only two of the eight strains analyzed revealed this sequence, perhaps because the strains were passaged through monolayer culture before sequence analysis. The fitness of CIs in HAE and cotton rats suggests that these two models represent growth in humans and contain the requisite protease for cleavage activation of authentic F, different from the enzyme that mediates cleavage of laboratory strain F in monolayer cells. Future studies will address the requirements for cleavage activation of F in natural host tissue.

The striking finding of a difference at the F cleavage site between laboratory strains and our clinical isolates is consistent with our other findings that the properties compatible with fitness *in vivo* result in viruses that are not viable in immortalized cells. In fact, each property of HN and F is notably different between the laboratory strains and the CI. The receptor avidity of the HNs derived from CIs is lower than that of HNs in the laboratory strains, already reducing the likelihood of fusion activation at the very first step of the entry process. The CI HNs also have a higher neuraminidase activity (from 4- to 10-fold higher), presumably reducing the length of receptor contact and thereby also reducing fusion activation. Each CI HN bears N556, while the laboratory strain HNs all bear D556. Expression of a laboratory strain HN with the single D556N mutation results in an almost 5-fold increase in neuraminidase activity. While several other residues may contribute to this phenotype, this individual residue, conserved among all CIs we studied, appears to be a significant contributor to the phenotype.

Although other specific sequences in HN and/or F differ between different CIs, we note that the CI fusion machineries behave similarly, perhaps with diverse strategies for attaining the same effect of lower net fusogenicity and decreased F activation. Activation of fusion by the CI HN/F pairs is greatly reduced in comparison to that in the laboratory strain HN/F pairs (Fig. 5C), and the F of CI viruses is itself less readily activated (Fig. 5B) (6). This stability of the CI F proteins in the prefusion state—compared to that of the laboratory strain F proteins—is not associated with differences in the heptad repeat regions, whose association is required for fusion. The stability of CI F proteins in their prefusion state thus cannot be due to a reduced association between HRC and HRN but instead may be attributable to a higher energy of activation for the transition of F from its metastable prefusion state to its postfusion state.

Each of these functional differences between the CI and laboratory strains contributes to a phenotype of less active fusion activation in the CI viruses and likely contributes to the difference in fitness *in vivo.* The striking preservation of these features in our panel of CIs suggests that mitigating fusion is important for infection of humans. The sequence evolution and functional results taken together suggest that mechanistic studies using viral strains that grow *in vivo*—in cells or tissues that represent the natural host—may reveal authentic viral properties important to infection and human disease.

## MATERIALS AND METHODS

### HPIV3 strains.

Clinical isolates from throat swab material were provided by Stephen Jenkins, Clinical Microbiology Laboratory at Weill Cornell Medical College, New York, NY. The isolates were supplied as a deidentified aliquot of the original sample material. Genome sequences were determined for HPIV3 clinical isolate strains referred to as CI-7, CI-8, CI-9, CI-10, CI-11, CI-12, CI-13, and CI-14. The laboratory-adapted (reference) strain of HPIV3 (Wash/47885/57) was obtained from the NIH (HA-1, NIH no. 47885, catalog no. V323-002-020) (34) and propagated and the titer determined on CV-1 cells as previously described (6).

### HAE cultures.

The EpiAirway AIR-100 system (MatTek Corporation) consists of normal human-derived tracheo/bronchial epithelial cells that have been cultured to form a pseudostratified, highly differentiated mucociliary epithelium closely resembling that of epithelial tissue *in vivo*. Upon receipt from the manufacturer, HAE cultures were transferred to 6-well plates (containing 0.9 ml medium per well) with the apical surface remaining exposed to air and incubated at 37°C in 5% CO_2_.

### Viral propagation in HAE.

HAE cultures were infected by applying 200 µl of EpiAirway medium containing 4,000 PFU of reference or other strain of HPIV3 to the apical surface for 90 min at 37°C. At 90 min, the medium containing the inoculum was removed, and cultures were placed at 37°C and fed each day with 0.9 ml medium via the basolateral surface. Viruses were harvested by adding 200 µl medium per well to the HAE cultures’ apical surface and allowed to equilibrate for 30 min at 37°C. The suspension was then collected, and viral titers were determined as previously described (8). This viral collection was performed sequentially with the same wells of cells on each day postinfection.

### Preparation and sequencing of HPIV3 RNA.

Clinical isolates, along with laboratory-adapted virus and three viruses with known mutations, were all grown in HAE cells. RNA was extracted and sequenced from extracellular supernatant fluid. Viral titers in cell extracts and supernatant fluid were estimated by quantitative PCR (qPCR). Viral RNA was extracted and processed with the NuGen Ovation amplification system v2 as previously described (35) with minor modifications. In all cases, 5 µl of total RNA was used as input for the NuGen kit (total RNA from the supernatant samples could not be quantified). Sequencing libraries were prepared using the Illumina TruSeq PCR-free sample preparation kit, with 2 µg cDNA input, and shearing was performed on the Covaris LE220 with the following settings: a duty factor of 15%, Peak Incident Power of 450, and 200 cycles per burst for 75 s. Libraries from all samples were sequenced on a single MiSeq run with 2× 300-bp paired reads.

### Genome assembly.

Reference-guided genome consensus assemblies were generated for each sample by aligning the sequencing reads to HPIV3 strain 14072 (GenBank accession no. EU424062.1) using BWA-MEM v0.7.5a-r405 (36), removing multimapped reads, and resolving conflicts using a majority rule. (Priority was given in the order A > C > G > T in the event of a tie.) Sequence coverage was determined using the SAMtools depth tool v1.1 (37).

### Time series analysis.

Five clinical isolates were sequenced on days 1, 3, and 7 after passage through HAE. In order to assess the impact of cell culture on the HPIV3 population, a time series analysis was performed. In particular, variants that changed in frequency as a function of time were identified. To accomplish this, for each clinical isolate, samples harvested from days 1, 3, and 7 were aligned to their respective day 1 consensus assembly. The number of reads mapping to each nucleotide base (A, C, G, T) at each position in the genome was extracted from the alignment files using bam-readcount v0.7.4 (38). The base frequencies were displayed as stacked histograms for each clinical patient for every genome position, and a Fisher exact test, extended to work with contingency tables larger than 2 by 2, was performed at each base in order to identify statistically significant shifts in nucleotide base frequencies (*P* < 0.01).

To confirm that the distance between isolates from different clinical patients was greater than the variability between isolates from different time points within the same patient, a Euclidean distance tree was generated. Specifically, a data matrix comprised of the frequency of each nucleotide base (A, C, G, T) at each position in the genome with at least 10× read coverage was considered (14,303 positions in total). A neighbor-joining tree was constructed using the Euclidean distances calculated from this matrix.

### Phylogenetic analysis.

The complete consensus sequences were aligned using MUSCLE, a program for creating multiple alignments of nucleotide sequences (39). PhyML was used to fit 28 models of DNA evolution (JC69, K80, F81, F84, HKY85, TN93, and GTR, each with or without “+I” and/or “+G”) (40). The model with the lowest Akaike information criterion (AIC) was selected as the best-fitting model. A maximum-likelihood tree was drawn in MEGA by using a general, time-reversible, gamma-distributed nucleotide substitution model (41). The reliability of the inferred tree was quantified with Felsenstein’s bootstrap test with 500 resamples.

### Minor variant detection.

In order to survey the genetic diversity within each sample, minority variant profiles were determined for each gene using FreeBayes v0.9.20-4, a Bayesian genetic variant detector designed to find single-nucleotide polymorphisms (SNPs) from high-throughput next-generation sequencing data (42). FreeBayes was configured to act as a frequency-based pooled variant caller, which identifies variants in terms of observation frequency rather than called genotypes. The following constraints were imposed on the FreeBayes algorithm: (i) at least 20 reads were needed to be mapped to a site in order to process it, (ii) at least 8 counts of observations supporting an alternate allele were required in order to evaluate the position, and (iii) at least 2% of observations were required to support an alternate allele in order to evaluate the position.

After variants were identified using FreeBayes, the variants were classified as either synonymous or nonsynonymous. For each gene, the consensus assembly nucleotide sequence was translated into a corresponding amino acid sequence. A copy of the consensus assembly nucleotide sequence was created, and, for each variant identified using FreeBayes, the identified variant replaced the original nucleotide at the variant’s specified location. This new nucleotide sequence was then translated into an amino acid sequence and was compared against the original amino acid sequence that results from the translation of consensus assembly. If the comparison resulted in identical amino acid sequences, the variant was marked as “synonymous”—otherwise, it was marked as “nonsynonymous.”

### Testing positive selection and correlation.

To evaluate the action of selective pressure on each of the coding regions among the HPIV3 strains, the rates of synonymous (*dS*) and nonsynonymous (*dN*) changes at amino acid sites were estimated using three different methods. Codeml, as implemented in the PAML suite, was used to identify positively selected sites with the Bayes empirical Bayes method (43). The single likelihood ancestor counting (SLAC) and fixed effects likelihood (FEL) methods as available on the Datamonkey webserver (http://www.datamonkey.org/) were used to assess the consistency of sites identified as having undergone positive selection (44).

### HN and F constructs and transient expression.

HPIV3 HN and F genes from laboratory strains and clinical isolate viruses were cloned into pCAGGS mammalian expression vectors. Transfections were performed with Lipofectamine 2000 according to the manufacturer’s protocols (Invitrogen).

### Assay of neuraminidase activity.

Monolayers of 293T (human kidney epithelial) cells transiently expressing lab-adapted or CI HNs were washed once with phosphate-buffered saline (PBS) and then incubated for 10 min in 5 mM EDTA in 1× PBS placed in pH 5.0 CO_2_-independent medium (Gibco). 2-(4-Methylumbelliferyl)-α-d-*N*-acetylneuraminic acid sodium salt (MUNANA [Toronto Research Chemical]) at 20 mM was added at 1:1 vol/vol for a final concentration of 10 mM MUNANA. A kinetic reading at 37°C was done using SpectraMax M5 enzyme-linked immunosorbent assay (ELISA) multimode reader.

### Assay of receptor avidity.

Partial receptor depletion of red blood cells (RBCs) was done as described in reference 10. RBCs were partially depleted of sialic acid present on the cell surface and then used to determine the relative receptor-binding avidities of variant HNs as described in references 8 and 10.

### β-Galactosidase complementation-based fusion assay.

We use an assay that we previously developed to detect early stages of fusion activation, performed as previously described in references 8 and 10.

### Ethics statement.

All animal work with HPIV3 was performed in the laboratory animal facility at the Ohio State University (OSU). OSU is accredited by the Association for Assessment and Accreditation of Laboratory Animal Care (AAALAC). All research involving animals was conducted in strict accordance with the *Guide for the Care and Use of Laboratory Animals* (45). The animal use protocol was approved by the Institutional Animal Care and Use Committee at OSU (Animal Use Protocol 2009A0183-R1 [approved 6 September 2012]).

### Animals, infection, and virus titration.

Inbred cotton rats were obtained from Harlan (Indianapolis, IN). Female animals 6 to 10 weeks of age were infected as previously described (8, 22).
